# Host phylogeny and microbiome composition predict gut nematode community composition within a diverse assemblage of African herbivores

**DOI:** 10.1098/rsbl.2026.0248

**Published:** 2026-08-26

**Authors:** Joel O. Abraham, Reed Leventis, Georgia Titcomb, Andrew Dobson, Andrew H. Moeller, Andrea Graham

**Affiliations:** 1 Department of Ecology and Evolutionary Biology, Princeton University, Princeton, NJ, USA; 2 Fish, Wildlife, and Conservation Biology Department, Colorado State University, Fort Collins, CO, USA

**Keywords:** African savanna, co-transmission, diet, *e*DNA metabarcoding, immune trade-offs, large mammalian herbivores, microbiome, nematodes, pathogenic bacteria, self-medication

## Abstract

Gut nematodes influence animal health and fitness, with effects shaped by their abundance and community composition. While closely related host species tend to harbour similar nematodes, the relative roles of host ecology and phylogeny in structuring nematode communities remain unclear. Here, we assess how host holobiont traits—body size, diet, space use, potentially pathogenic gut microbes and overall gut microbiome composition—predict gut nematode abundance and composition, while controlling for host phylogenetic relatedness. We jointly analyse DNA metabarcoding data on nematodes, microbiomes and diets from 17 free-ranging African herbivore species. Host phylogeny and microbiome composition were the strongest predictors of nematode community structure. Physical proximity and diet also contributed, although to a lesser extent, whereas body size did not. Nematode abundance correlated positively with the richness of putative pathogenic bacteria, which in turn increased with diet richness. Nematode presence/absence covaried with the microbiome and diet composition, and we identified pairwise associations between nematodes, putative pathogenic bacteria and diet plants. Our findings illustrate that host ecology and phylogeny jointly influence gut nematode communities. In particular, the gut microbiome is a key predictor of nematode communities, even after accounting for host phylogeny, emphasizing the ecological interconnectedness of these gut constituents.

## 1. Introduction

Parasitic gut-dwelling nematodes affect animal health, fitness and population dynamics [1,2]. Their effects depend on their abundance and species composition: heavier infections typically cause greater harm, and nematode species differentially impact hosts [2–6]. Previous research in free-ranging herbivores has shown that gut nematode composition is shaped by host species identity and phylogenetic history [7–10]: while many nematodes can infect multiple host species [7,11,12], host species tend to harbour distinctive parasite communities, and closely related species support more similar nematode assemblages [7–10]. However, predictors of variation in nematode community composition across host species remain poorly understood [4,7,11].

Several phylogenetically structured host traits—e.g. body size, diet, space use, pathogenic microbes that they harbour and microbiome structure overall—have been proposed as potential drivers of nematode community structure (the ‘nemabiome’). Body size might influence the nemabiome because larger animals, with larger guts and greater food intake, might support more or larger nematodes, or greater nematode diversity [13–18]. Body size also affects behaviour, including what and where animals eat [19–21], which, in turn, could influence parasite exposure through co-transmission, in which nematodes and microbes are obtained from the same sources [8–10,18,22–26]. Diet might further shape nematode communities via self-medication: some plants have antiparasitic properties, so animals that consume medicinal plants may exhibit lower nematode burdens [27–29].

Microbiome composition may also influence nematode communities [30–32], either through direct nematode–microbial interactions—consumptive, competitive or facilitative (e.g. the secretion of antihelminthic or antimicrobial compounds [33])—or indirectly via host immunity [33–37]. For example, the immune system’s Th1 branch primarily targets microparasites (e.g. bacteria, viruses), while suppressing the Th2 branch that targets macroparasites (e.g. nematodes) [34]. Trade-offs between Th1 and Th2 responses may drive trade-offs between microparasite and microparasite loads [33–35]. Hosts also differ in overall immune investment, which may generate covariation in susceptibility to both pathogenic bacteria and nematodes [36,38]. Finally, these host–nematode–microbe relationships may depend on nematode traits such as size and feeding habit [2–4]; for instance, blood-feeding nematodes particularly suppress host immunity (e.g. [39]) and may thus facilitate microbial proliferation.

We aimed to assess the contributions of body size, diet, space use, bacterial pathogen load and microbiome composition to nemabiome structure. Because these hypothesized predictors are phylogenetically structured and interrelated [8,10,13,14,19,40–43], it is critical to assess associations in a way that controls for host phylogeny and isolates individual factors. Here, using faecal DNA metabarcoding data on nematodes, diets and microbiomes from a diverse community of free-ranging African savanna herbivores, we expand on prior work [7,40,44] to investigate how these factors shape gut nematode abundance, diversity and composition. By integrating previously published datasets within a unified analytical framework, we enable direct comparison of the contributions of different factors. In so doing, we present a reproducible, phylogenetically informed approach that can be applied and extended in future studies of complex ecological communities.

## 2. Material and methods

### (a) DNA metabarcoding data

DNA metabarcoding data on diet, microbiome and gut nematodes were generated from faecal samples collected between 2013 and 2017 in Laikipia County, Kenya [7,40,44]. Across datasets, data were available for 17 herbivore species (14 wild, three domestic): from these species, 1075 samples were analysed for diet (*trnL*-P6 marker), 354 for microbiome (16S-V4 rRNA marker) and 266 for nematode composition (ITS-2 rRNA marker) [7,40]. An additional 457 samples were tested for nematode presence via qPCR; primarily those that tested positive for nematodes (qPCR cycle threshold [*C_t_*] < 35) were sequenced for nematode composition [7]. All data types were available for 87 samples (supplementary material, table S1).

Data were generated via Illumina sequencing and processed using consistent pipelines [7,40,44,45] (see supplementary material for further detail). Sequences were then clustered into mOTUs for diet (*n* = 213) and nematode data (*n* = 96; supplementary material, table S2), and into ASVs for microbiome data (*n* = 29 308) [46].

### (b) Identifying potentially pathogenic bacteria

We hypothesized that, because of their negative effects on host fitness, pathogenic bacteria might exhibit especially strong interactions with hosts and nematodes [34,35,47]. To identify taxa with pathogenic potential, we used the 16SPIP bioinformatics pipeline on 347 samples for which raw microbiome sequence data were available [48]. 16SPIP employs a reference database constructed from 29 258 16S reads covering 346 bacterial species classified as of human health concern by China’s Ministry of Health [48]. Although 16SPIP’s reference database is not exhaustive [49,50] and is biased toward pathogens of humans and domesticated animals [48], this approach enables the consistent identification of bacterial taxa with established pathogenicity in large mammals from short-read 16S data. Importantly, 16SPIP annotations do not constitute functional confirmation of pathogenicity within the present host species, but rather bacteria with documented pathogenic potential in prior literature. 16SPIP identified 144 species categorized as potentially pathogenic (hereafter, ‘putative pathogenic bacteria’) in our data [46].

### (c) Assessing the compositional effects of herbivore species identity

**Figure 1.**
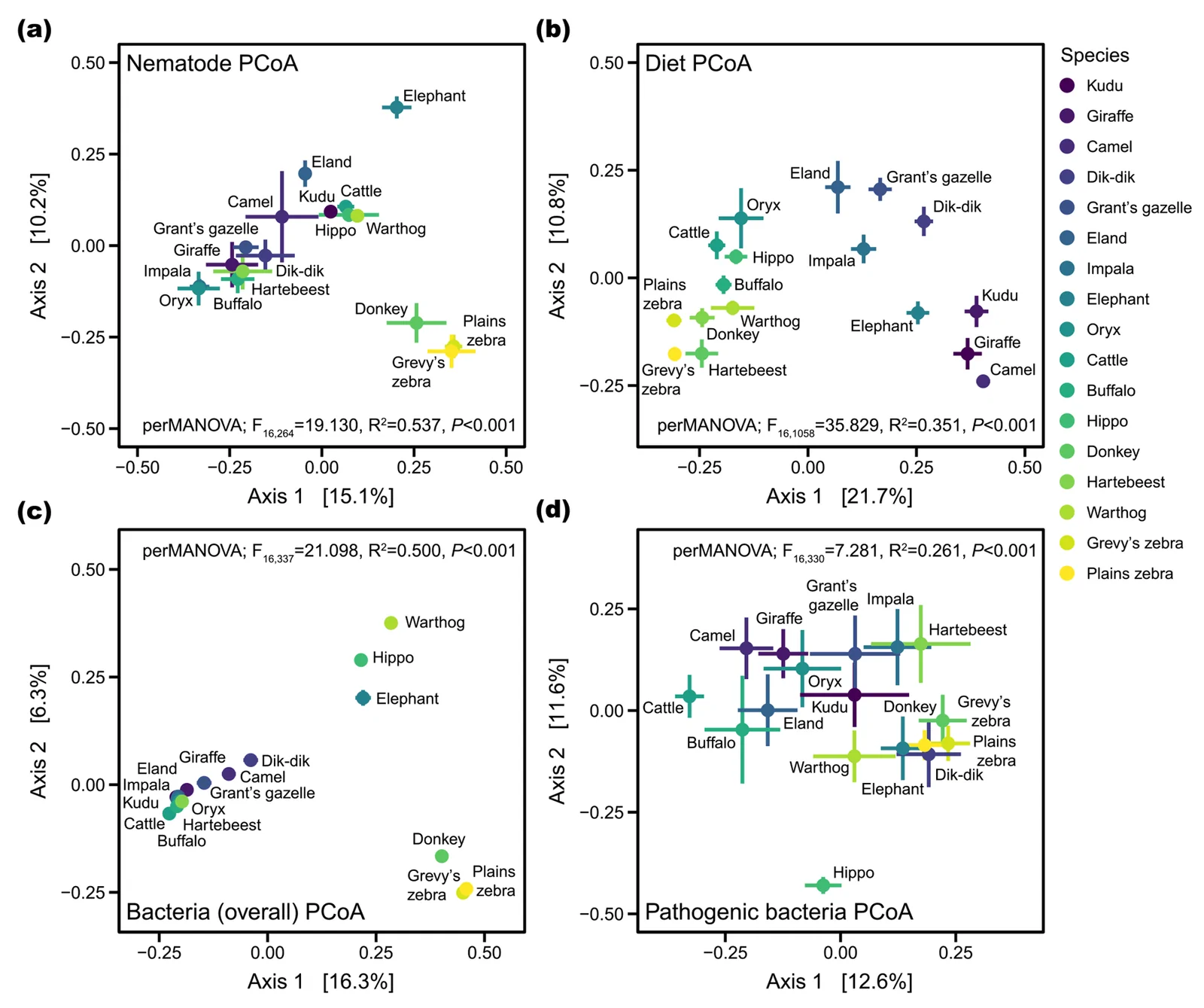
Herbivore species identity is a primary driver of compositional variation. PCoA ordination plots visualizing Bray–Curtis dissimilarity in (a) nematode, (b) diet, (c) overall bacterial and (d) putative pathogenic bacterial community composition. Points represent species centroids. Bars represent 95% confidence intervals. Point and bar colours correspond to herbivore species. Statistics from perMANOVAs evaluating the significance of herbivore species identity in structuring community composition are shown in each panel. In all cases, herbivore species identity significantly predicted compositional variation.

**Figure 2.**
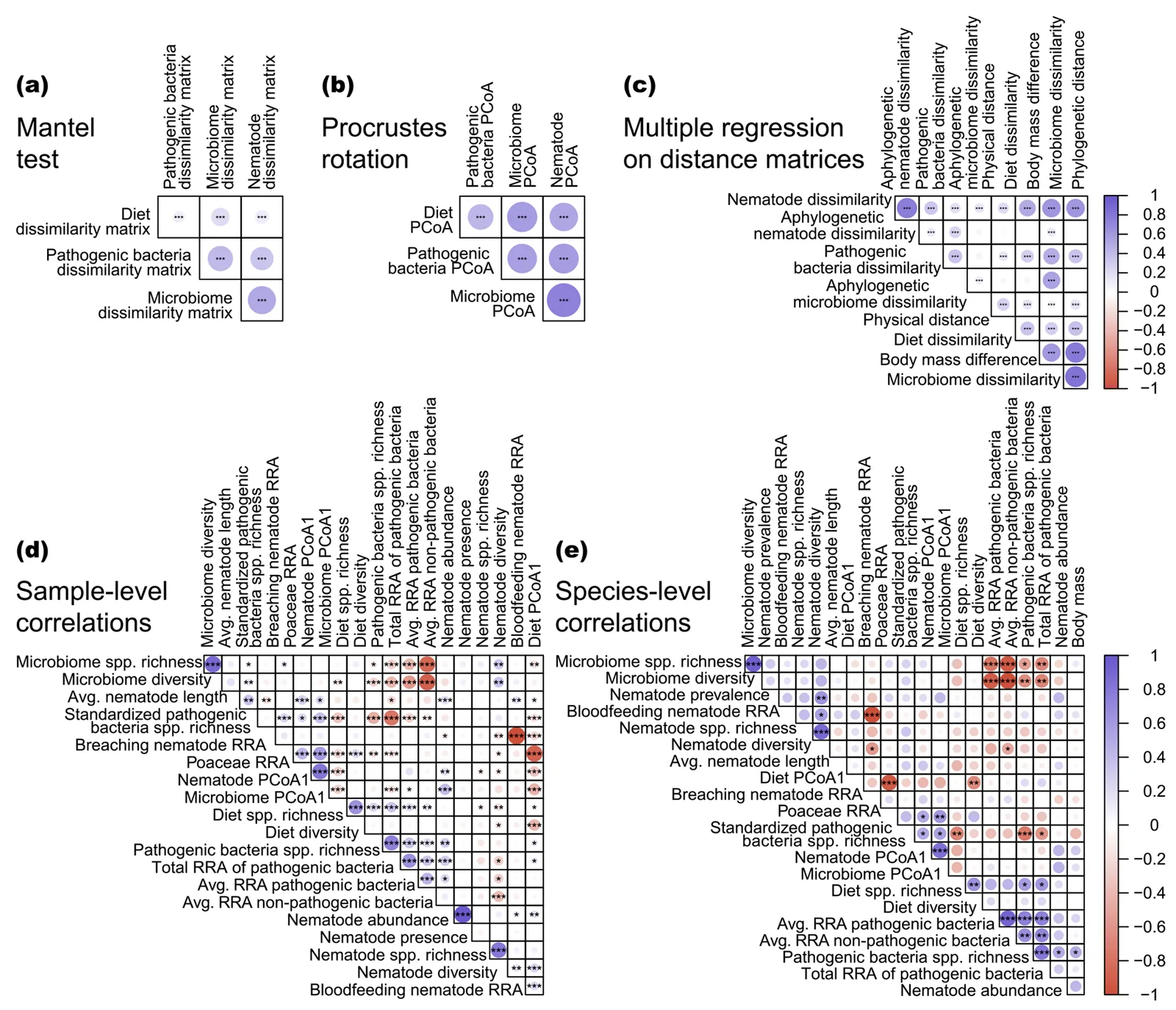
Pairwise correlations across datasets. (a) Correlations from Mantel tests, reflecting the degree to which Bray–Curtis dissimilarity matrices are correlated. (b) Correlations from Procrustes rotation analyses, reflecting the degree to which PCoA ordinations are transposable. (c) Pairwise correlations resulting from multiple regression on distance matrices between Bray–Curtis dissimilarity matrices and potential predictors of compositional variation. Correlation plots in (d) and (e) depict (d) sample-level and (e) species-level (aphylogenetic) relationships between aggregate properties of datasets. Circle colours and sizes show the direction and magnitude of each correlation (per the gradients on the right). Asterisks indicate statistical significance at different thresholds (* *p* < 0.05; ** *p* < 0.01; *** *p* < 0.001). Variables are ordered by similarity, according to hierarchical clustering.

All analyses were conducted in R v. 4.3.2 [51]. To assess compositional concordance across diet, nematode, microbiome and pathogenic microbe datasets, we calculated Bray–Curtis dissimilarities between samples and visualized patterns using PCoA with ‘vegan’ [52] (figure 1; supplementary material, figure S1). Next, we subsetted each pair of datasets to overlapping samples and quantified compositional similarity using Mantel tests on Bray–Curtis matrices and symmetric Procrustes analyses on PCoA ordinations to evaluate their transposability (figure 2a,b).

Given strong clustering by herbivore species in all datasets, we used permutational analysis of variance (perMANOVA), implemented in ‘vegan’ [52], to quantify the herbivore species effect. We also used pairwise perMANOVAs, implemented in ‘pairwiseAdonis’ [53], to evaluate compositional differences between species pairs within all four datasets (supplementary material, table S3), correcting the resulting *p*-values for multiple comparisons using the Benjamini–Hochberg false discovery rate (FDR) correction [54]. To identify the taxa driving herbivore species effects (supplementary material, tables S4–S7), we performed indicator species analyses using *multipatt* in ‘indicspecies’ [55], which calculates associations between each community member and herbivore species and evaluates their statistical significance via permutation tests (with 9999 permutations). We again adjusted *p*-values for multiple comparisons with the Benjamini–Hochberg FDR correction [54].

To further identify taxa in each dataset responsible for herbivore species effects, we conducted co-occurrence analysis using ‘cooccur’ [56], determining significant associations between taxa (after correcting for multiple comparisons [54]) based on their co-occurrence within samples (supplementary material, table S8). For samples overlapping across all four datasets (*N* = 87), we merged RRA-by-sample matrices, converted them to presence/absence (RRA > 0 = present) and visualized significant associations using ‘igraph’ [57] (supplementary material, figure S2). We excluded full microbiome data owing to the limited taxonomic resolution and the large number of ASVs, which made inclusion both interpretively and computationally infeasible.

### (d) Weighting factors predictive of nematode composition

To assess predictors of nemabiome composition, we used multiple regression on distance matrices (MRM). We first quantified candidate predictors of pairwise nemabiome Bray–Curtis dissimilarities (figure 2c). To assess associations with body size, we calculated pairwise differences between species’ body masses using data from COMBINE [58], a mammal trait database compiled from primary sources. To assess phylogenetic relationships, we computed species-pair phylogenetic distances from a mammalwide phylogeny [59], using *cophenetic* in ‘ape’ [60]. To evaluate physical proximity (and potential for co-transmission), we calculated pairwise distances between sampling locations using *st_distance* in ‘sf’ [61]. To normalize distributions, these predictors were log-transformed after adding 1. Because microbiome composition exhibits strong phylogenetic signal [41,62,63], we additionally derived an ‘aphylogenetic’ microbiome dissimilarity metric: we regressed microbiome dissimilarity against phylogenetic distance using MRM, implemented in ‘ecodist’ [64] and used the resulting residuals as phylogeny-independent microbiome dissimilarities. All predictor matrices were scaled to range from 0 to 1 to facilitate comparisons of predictive strength.

We then fit MRM models with nematode Bray–Curtis dissimilarities as the response and the following predictors: body mass difference, phylogenetic distance, geographic distance, diet dissimilarity, pathogenic microbiome dissimilarity and overall microbiome dissimilarity (phylogenetic or aphylogenetic) (figure 2c; supplementary material, table S9). Predictor significance was assessed using permutation tests with 1000 permutations.

Analyses were restricted to the 87 samples present across all datasets. Because sample sizes were small for some host species (six species with three or fewer samples; supplementary material, table S1), we conducted a sensitivity analysis in which all samples from each host species were iteratively excluded and models were refit; we evaluated the consistency of results across data subsets to assess the influence of individual host species on results (supplementary material, table S9). All analyses were conducted using both phylogenetic and aphylogenetic versions of microbiome dissimilarity.

For completeness and comparability, we applied the same analytical framework to the other datasets (supplementary material, table S9; see supplementary material for additional details).

### (e) Assessing diversity and abundance relationships

We also examined relationships between diversity and abundance across datasets (supplementary material, table S10; see supplementary material for descriptions of variables calculated). We assessed pairwise linear correlations between variables at both sample and species levels (figure 2d,e). For relationships significant at both scales (*p* < 0.10), we tested within-species significance using linear mixed-effects models (species as a random effect) in ‘lme4’ [65]. We also tested species-level associations while controlling for phylogeny using phylogenetic linear models implemented in ‘phylolm’ [66], employing the same phylogeny as above [59].

### (f) Identifying nematode–microbes and nematode–diet associations

We identified plant and bacterial taxa associated with nematodes using the aforementioned co-occurrence analysis [56]. To assess relationships between nematode presence/absence and composition within each dataset, we ran stratified perMANOVAs (stratified by herbivore species) on samples in diet, microbiome and putative pathogenic bacteria datasets with corresponding nematode data [52]. We also used indicator species analyses to identify taxa uniquely associated with nematode presence/absence (supplementary material, tables S11–S13) [55].

## 3. Results

### (a) The effect of herbivore species identity

In all four datasets, samples clustered strongly by herbivore species (figure 1; supplementary material, figure S1), which explained 26.1–53.7% of compositional variation: 26.1% for putative pathogenic bacteria, 35.1% for diet, 50.0% for microbiome and 53.7% for nemabiome. Most herbivore species exhibited unique associations across all datasets (supplementary material, tables S4–S7). For nematodes specifically, 76 of 96 mOTUs (79.2%) were uniquely associated with particular host species; 16 of 17 herbivore species (all except camel) exhibited distinct nematode associations, ranging from 1 (oryx, hippo, giraffe) to 19 mOTUs (elephant; supplementary material, table S7). Co-occurrence analysis also demonstrated strong host-specificity, identifying 13 direct links between hosts and nematodes, all positive (mOTUs more frequent than expected by chance; supplementary material, figure S2 and table S8).

Resultantly, nematode community composition was strongly correlated with diet, putative pathogenic bacteria and overall microbiome composition: Bray–Curtis dissimilarity matrices were significantly correlated, such that sample pairs dissimilar in one dataset tended to be dissimilar in others (figure 2a), and PCoA ordinations were highly transposable (figure 2b).

Despite high cross-dataset concordance overall, samples clustered differently within datasets (figure 1; supplementary material, figure S1 and table S3). For example, equids consistently clustered together but were most distinctive in nematode and microbiome ordinations. Warthog and hippo clustered with elephants in microbiome data but with ruminants in nematode data, while elephants remained distinct.

### (b) Factors predictive of nematode community composition

Microbiome dissimilarity, phylogenetic distance, physical distance and diet dissimilarity were all significant predictors of nemabiome composition when the full dataset was analysed (with either phylogenetic or aphylogenetic versions of the microbiome variable; supplementary material, table S9). Collectively, these predictors explained 44.5% of the variation in nemabiome dissimilarities. Microbiome dissimilarity and phylogenetic distance were statistically significant in all models and consistently exhibited strong positive effects, generally more than twice the magnitude of other predictors. Physical distance and diet dissimilarity were significant in most models (34/36 and 27/36, respectively) and had effects of consistent direction: geographic distance was positively and diet dissimilarity was negatively associated with nemabiome dissimilarity. Despite limited sample sizes for some host species (supplementary material, table S1), results were qualitatively robust to the exclusion of each species.

### (c) Diversity and abundance relationships

**Figure 3.**
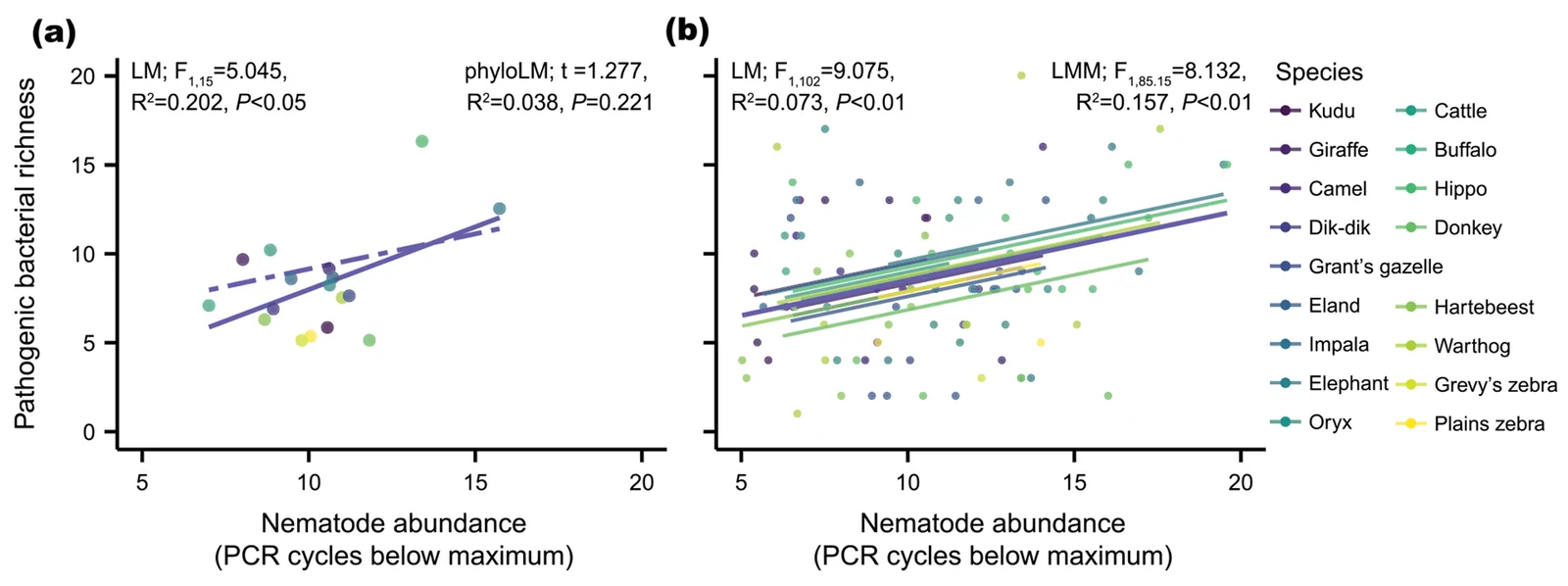
Nematode abundance increases with the richness of potentially pathogenic microbes. (a,b) Putative pathogenic bacterial richness increased with nematode abundance. Panel (a) depicts species-level relationship, wherein points correspond to within-species means, while panel (b) depicts corresponding sample-level relationship, wherein points represent individual samples. Points are coloured by species identity. The solid blue line in (a) corresponds to aphylogenetic regression (LM), while the dashed blue line corresponds to the equivalent phylogenetic regression (phyloLM). Thinner coloured lines in (b) correspond to a linear mixed-effects model (LMM) including random intercepts for species and are coloured by species identity. Statistics from all models are shown in the corresponding panels.

Nematode abundance was positively associated with putative pathogenic bacterial richness, a relationship that held both across and within species (figure 3a,b), although the species-level relationship was not significant after accounting for host phylogeny. While no other factors exhibited relationships with nematode abundance, diversity or traits consistently significant across scales (figure 2d,e; supplementary material, table S10), several patterns emerged for putative pathogenic bacteria: both pathogenic bacterial richness and relative abundance increased with diet richness, associations robust across scales (but not to phylogenetic non-independence; supplementary material, figure S3a–d). Furthermore, the relative abundance of putative pathogenic bacteria declined with increasing microbiome diversity, a pattern consistent across scales and independent of phylogenetic relatedness (supplementary material, figure S3e,f), although one potentially reflective of compositionality constraints on microbiome data.

### (d) Nematode–microbe and nematode–diet associations

Consistent with strong associations between microbiome and nematode composition documented above, nematode presence/absence significantly predicted variation in both overall microbiome (perMANOVA; *R*^2^ = 0.011, *F*_1,144_ = 3.249, *p* < 0.001) and putative pathogenic bacteria composition (perMANOVA; *R*^2^ = 0.017, *F*_1,142_ = 3.214, *p* < 0.001) after controlling for host identity. Of 29 308 bacterial ASVs, 110 were linked to nematode absence, and two were linked to nematode presence (supplementary material, table S11). Two putative pathogenic taxa, *Streptococcus orisratti* and *S. suis*, were more frequent in the absence of nematodes (supplementary material, table S12). From co-occurrence analysis, we recovered 25 associations between nematodes and putative pathogenic bacteria, 24 positive and one negative (supplementary material, figure S2 and table S8).

Although diet had weaker associations with nematode composition, nematode presence/absence and diet composition likewise co-varied (perMANOVA; *R*^2^ = 0.006, *F*_1,144_ = 4.086, *p* < 0.001), with 12 of 213 plant mOTUs significantly associated with nematodes (11 with absence and one with presence; supplementary material, table S13). Diet composition correlated not only with whether any nematodes were detected but also with the species of nematodes detected: there were 83 pairwise links between forage plant and nematode mOTUs, 76 positive and seven negative (supplementary material, figure S2 and table S8).

## 4. Discussion

In this study, we used hypothesis-driven, integrative analyses of observational data to assess predictors of nemabiome structure in a diverse assemblage of African herbivores. Using DNA metabarcoding data from 17 host species, we quantified associations among body size, diet and microbiome composition and among nematode abundance, diversity and composition within large mammalian herbivores, controlling for effects of host phylogeny. In so doing, our results address long-standing hypotheses in parasite ecology, supporting some (outlined below) while recovering no evidence for others (including roles for nematode traits and host body size).

First, host species identity predicted nematode, microbiome and diet composition, playing an especially dominant role in structuring the nemabiome. While prior work also documented effects of host identity on nematode composition [7–10], this remains somewhat surprising given that the diversity of hosts in this system might be expected to select for generalist nematodes [11,67–69]. Yet nearly 80% of nematode taxa were associated with a particular host species, suggesting highly specific, probably co-evolved, relationships [7,12,26,70,71]. This level of specialization has important ecological implications: nematode diversity may track host diversification, and host declines could trigger parasite loss through co-extinction [72–75].

Second, host phylogenetic relatedness and microbiome composition (independent of phylogeny) emerged as primary predictors of nemabiome structure. The strong association with microbiome composition irrespective of host phylogenetic relatedness is consistent with a central role of the microbiome in shaping nematode assemblages and vice versa [30–32]: nematode presence was significantly related to both overall microbiome and putative pathogenic bacteria composition; nematode abundance increased with putative pathogenic bacterial species richness; and diverse pairwise associations emerged between individual nematode species and putative pathogenic bacteria. These results imply that multiple mechanisms mediate covariation between nematodes and bacteria. Negative associations might result from competition for host resources [30,31], immune trade-offs (e.g. Th1 versus Th2 immunity [33–35]) and direct nematode–bacteria interactions (e.g. predator–prey interactions), whereas positive associations, which were most common in our analyses, may reflect facilitation (via, e.g. immunosuppression [33,37]) or co-transmission [22–25]. Future research might disentangle these mechanisms by focusing on specific taxa. For instance, both *Streptococcus orisratti* and *S. suis* were more common in the absence of nematodes and uniquely affiliated with warthogs; yet they exhibited a positive association with the nematode *Oesophagostomum dentatum*, likewise positively associated with warthogs. These patterns imply that host species context may partially mediate nematode–microbe interactions and suggest *Streptococcus* spp. as promising candidates for mechanistic studies into their pathogenicity and interactions with nematodes within wild herbivores.

Third, diet was subtly related to nemabiome composition. Nematode presence/absence weakly predicted diet composition, and several plant species were significantly associated with particular nematodes (as well as putative pathogenic bacteria). These patterns could reflect self-medication—with negative associations indicative of plants protective against parasites and positive associations indicative of plants that herbivores select while infected [27–29]—or parasite exposure owing to foraging in specific areas or consuming particular plants [22–25]. Despite limited available evidence of self-medication in wildlife, several plants associated with nematode absence here have documented medicinal properties [76–78] and some (e.g. seq_001382, *Achyranthes aspera*; seq_016960, *Vepris* spp.; supplementary material, table S13) are even used in traditional medicine to treat nematode infections [79,80]. Interestingly, after accounting for other factors, diet dissimilarity correlated negatively with nematode dissimilarity, indicating that even herbivores with distinct diets encounter similar parasites. That said, because diets vary considerably through time and our data represent a single timepoint [21,40,45], whereas nematode infections are typically more chronic [2,4], these data may underrepresent diet–nemabiome associations. Indeed, some host species are represented by relatively few samples in our analyses, limiting our power to infer associations. Relatedly, although multiple steps were implemented to minimize spurious linkages [7,40,44,45], we avoided excluding associations based on presumed biological plausibility, preserving the opportunity to identify previously undocumented interactions.

Finally, dietary richness correlated positively with putative pathogenic bacterial richness and relative abundance, contrary to common assumptions that diverse diets protect against pathogens [81–83]. While these bacterial taxa are not confirmed to be pathogenic to our focal host species, these patterns may indicate that consuming many plant species increases pathogen exposure opportunities, or that animals may broaden their diets in response to infection. In contrast, overall microbiome diversity correlated negatively with putative pathogenic bacterial abundance. This negative association could reflect compositionality constraints (less diverse communities necessarily exhibit higher relative abundances of individual taxa), but also accords with enhanced pathogen resistance in more diverse microbiomes [84–87].

## 5. Conclusion

Our results demonstrate that nematode composition closely mirrors host phylogeny and co-varies with diet, especially microbiome composition. These findings underscore the importance of the microbiome and diet for animal health [31,41,81,82], with consequences for parasite infection outcomes and host population dynamics [1,5,6,17,33,35,37]. More broadly, our results provide anchor points for future, targeted experimental work (e.g. with deworming and/or feeding trials) aimed at further disentangling the processes structuring parasite communities in the wild.

## Supplementary Material

Supp Material 1

Supp Material 2

Supplementary material is available online [90].

Supplementary material is available online at https://doi.org/10.6084/m9.figshare.c.8593776.

## Data Availability

All data and code are publicly available and cited in the text where relevant: diet and microbiome data are available in one Dryad repository [88] and nematode data are available in a second Dryad repository [89]. All data have also been consolidated into a single Dryad repository associated with this publication, along with the R scripts necessary to replicate all analyses [46].
