# Supplementary material for: Host phylogeny and microbiome composition predict gut nematode community composition within a diverse assemblage of African herbivores
Source: Biol Lett. Author manuscript; Available in PMC 2026 Sep 17. (PMC13584170; doi:10.1098/rsbl.2026.0248)
Supplement: Supplementary file 1 [file NIHMS2207758-supplement-Supp_Material_2.docx]

**SUPPORTING MATERIALS FOR**

**TITLE: Host phylogeny and microbiome composition predict gut nematode community composition within a diverse assemblage of African herbivores**

**AUTHORS:** Joel O. Abraham^1^, Reed Leventis^1^, Georgia C. Titcomb^2^, Andy P. Dobson^1^, Andrew H. Moeller^1†^, and Andrea L. Graham^1†^

**AFFILIATIONS**

^1^Department of Ecology and Evolutionary Biology, Princeton University, Princeton, New Jersey, United States

^2^Fish, Wildlife, and Conservation Biology Department, Colorado State University, Fort Collins, Colorado, United States

^†^These authors contributed equally.

**SUPPLEMENTARY MATERIALS AND METHODS**

*DNA metabarcoding data generation*

Fresh herbivore dung samples were collected between 2013–2017 at Mpala Research Centre and Ol Jogi Conservancy in Laikipia County, Kenya [7,40,44]. Together, these sites span ~440 km² of thorn-scrub savanna and support over 20 large herbivore species, including livestock [7,13,44].

Samples were stored at *ca.* 4°C for 1–4 hours prior to returning to the lab. Samples were then homogenized, and pea‐sized subsamples were transferred into tubes containing silica beads and a DNA stabilization buffer (Zymo Xpedition™ Stabilization/Lysis Solution; Zymo Reseach, CA, USA). Tubes were frozen (at −20°C) until transport to the United States. Before importation to the US, samples were subjected to antiviral treatment as mandated by the US Department of Agriculture. Upon arrival in the US, samples were stored at −80°C prior to DNA extraction. Fecal DNA was extracted using Zymo Xpedition™ Soil/Fecal DNA MiniPrep kits following manufacturer instructions in a dedicated facility at Princeton University (NJ, USA). Samples were processed in small batches (typically 30) with one extraction control (sample-free extract) per batch to enable the identification of any contaminants introduced during DNA extractions. PCR was then conducted on all samples, as well as on all extraction controls and negative PCR controls (nuclease-free molecular-grade water instead of DNA extract); for diet analysis only, positive PCR controls (DNA extracted from plants during the construction of a local plant barcode reference library [91]) were included as well. In all cases, PCR was conducted using tagged primers containing a unique combination of forward and reverse 8-base pair tags (each differing by at least 2 base pairs from all other tags) for each sample or control, so that samples were uniquely identifiable post-PCR. The results of all PCR reactions were visualized using gel electrophoresis to confirm DNA amplification [40].

Distinct primer sets targeting different regions of the genome were used in PCR to generate each dataset. For diet analysis, the P6 loop of the chloroplast *trn*L(UAA) region was targeted for amplification, using uniquely tagged primers *trn*L(UAA)g and *trn*L(UAA)h (*trn*L(UAA)g: 5’-GGGCAATCCTGAGCCAA-3’; *trn*L(UAA)h: 5’-CCATTGAGTCTCTGCACCTATC-3’) [13,44,45,92]. Because samples were uniquely indexed during amplification, PCR products were then pooled and cleaned using Zymo Clean and Concentrator kits. Resultant libraries were quality-checked and sequenced on the Illumina HiSeq 2500 Rapid Flowcell (Illumina, CA, USA) at Princeton University’s Lewis Sigler Institute as single-end 170 bp reads along with 7 bp index reads.

For microbiome analysis, the V4 hypervariable region of the 16S rRNA gene was amplified, using indexed Illumina primers (515F: 5’-GTGYCAGCMGCCGCGGTAA-3’; 806R: 5’-GGACTACNVGGGTWTCTAAT-3’) [13,40]. Samples were pooled into libraries which were then cleaned and concentrated using Agencourt AmpureXP magnetic beads. Cleaned libraries were validated and quantified using Qubit dsDNA HS, Caliper LabChipGX HS DNA, and Kapa Biosystems Illumina Library Quantification qPCR assays [40]. Libraries were sequenced on an Illumina MiSeq Flowcell (v2) at Princeton University’s Lewis Sigler Institute using a 500-cycle v2 reagent cartridge in a 2×250bp paired end format.

For nemabiome analysis, the ITS-2 region of the ribosomal RNA gene was amplified, using tagged NC1f and NC2r primers (NC1f: 5’- ACGTCTGGTTCAGGGTTGTT -3’, NC2r: 5’- TTAGTTTCTTTTCCTCCGCT - 3’) [7]. PCR products were pooled into libraries and purified using MinElute PCR purification kits (Qiagen, MD, USA). Libraries were then prepared for sequencing using a PCR-free approach with the PrepX DNA Library Kit (Takara Bio Inc.) and sequenced on an Illumina MiSeq Flowcell in paired-end format (2×300bp) at Princeton University’s Lewis Sigler Institute.

*DNA metabarcoding data processing*

Resultant sequences from all sequencing runs were then assigned to the samples from which they originated (*i.e.,* demultiplexed), screened for putative errors and contaminants, and assigned taxonomic classifications. Bioinformatics approaches differed between datasets.

For diet data [40], paired-end reads were aligned and merged using ‘OBITools’ [93], using the *illuminapairedend* command. Sequences were demultiplexed with the *ngsfilter* command with parameters allowing for zero tag errors and a maximum of two primer errors. To eliminate erroneous sequences, sequences <8 bp, those with ambiguous nucleotide bases, or those with mean Illumina fastq quality scores ≤30 were discarded. For remaining sequences, the *obiuniq* command was used to group identical sequences and tally them within samples; singletons (*i.e.*, sequences that occurred only once) were removed. Next, PCR artifacts were discarded using the *obiclean* command: a sequence was considered to be a probable PCR artifact if it was highly similar to another sequence (1 bp difference) and had 5% of its reads in samples where both sequences occurred. Remaining sequences were matched against two reference libraries, both (*i*) a near-comprehensive local reference library containing 460 of the roughly 500 plants present at Mpala [91] and (*ii*) a global reference library compiled from the European Nucleotide Archive (release 130), using the *ecoTag* command. Sequences that did not perfectly match a sequence in the local and/or global reference libraries were then removed as a further precaution against chimeras, PCR artifacts, and sequencing errors. When a sequence perfectly matched a different species in each reference library, the identification based on the local library was preferentially retained. All remaining sequences were designated as molecular operational taxonomic units (mOTUs).

For microbiome data [40], sequence data were processed using ‘DADA2’ [94], according to the amplicon sequence-variant (ASV) approach. This approach filters the raw Illumina sequence data and also enables taxonomic assignments, resulting in an output matrix of ASVs within samples, where the ASVs represent bacterial taxa [40]. Within ‘DADA2’, primers were removed from the forward and reverse sequence reads using the *trimLeft* and *truncLen* commands. Terminal portions of each read were trimmed following any base with an Illumina quality score ≤2, and all sequences containing ambiguous nucleotide bases were discarded. The maximum number of expected errors allowed (maxEE) was set to the default value of 2 for both forward and reverse reads. Sequencing error patterns were identified using the *Learnerror* function in ‘DADA2’; the resultant parameters, together with the ‘DADA2’ sequence-variant algorithm, were used to denoise and dereplicate forward and reverse sequence reads. The output of this algorithm was a set of forward and reverse sequence-variants that were then merged into a table of sequence counts. The dataset was then screened for chimeras, which were removed using the *removeBimeraDenovo* command. Taxonomic identities were assigned to all remaining sequences using the GreenGenes v.13.8 reference database of microbial 16S rRNA sequences [95]: first, all sequences that occurred between primer pairs were extracted from the reference database and truncated to the expected sequence length (213 bp). Then the extracted reads and reference taxonomy were used to train the ‘QIIME2’ multinomial naïve Bayes classifier [96]; the *classify-sklearn* function was then used to identify ASVs based on the default confidence level for classification (>0.7). Sequence variants identified as Archaea, chloroplast, and mitochondria were removed, along with those that were unassigned at the kingdom level, leaving only ASVs that were classified in the kingdom Bacteria.

Nematode data [7] were, like diet data, processed using ‘OBITools’ [93], and the bioinformatics approach was therefore similar. Again, paired-end reads were aligned and merged using the *illuminapairedend* command; sequences with a low alignment-quality score (<40, the value corresponding to perfect alignment between the last 10 bases of each read) were discarded. Sequences were then demultiplexed using the *ngsfilter* command. Identical sequences were merged and tallied using *obiuniq.* Low-quality sequences were removed, which for nematode data were those with ambiguous nucleotide bases, those shorter than 100bp (expected length ranged from 300-450bp), and sequences represented by fewer than 10 reads. Taxonomic assignment was then made, first using *ecoTag* to match sequences with a reference database derived from in the EMBL genetic database (release 134; [97]) by *in silico* PCR (ecoPCR program; [98]); sequences not assigned to the Nematoda phylum with an assignment score of >70% similarity were discarded. Then, to improve taxonomic assignments, the nemabiome database (https://www.nemabiome.ca/; [99]) was downloaded and the *assignTaxonomy* function from ‘DADA2’ [94] was used to identify sequences, with a minimum bootstrap support of 80. Sequences were then clustered into mOTUs based on a 98% similarity threshold using ‘SUMATRA’ for alignment [100] and ‘MCL’ for clustering [101]; the most abundant sequence within each cluster was taken to be representative of that cluster. Note that processed data are also available using a 99% similarity threshold [7]. Probable contaminant sequences (*i.e.,* sequences more prevalent in negative controls than in samples) were eliminated using the ‘microDecon’ R package with default parameters [102].

Finally, to account for differences in sequencing depth among samples, sequence reads per sample were rarefied to the minimum number of reads per sample across all samples within a dataset: diet data were rarefied to 1293 reads per sample [40,44], nematode data were rarefied to 748 reads per sample [7], and microbiome data were rarefied to 2643 reads per sample [40]. Rarefaction curves were generated and visually inspected to verify that taxon accumulation with increasing read depth had reached an asymptote at the specified read thresholds, indicative of adequate sequencing depth. For all three datasets, the read depths to which data were rarefied were sufficient to capture this asymptote [7,40,44]. After rarefaction, data were converted into relative read abundances (RRA) by dividing the number of reads of each sequence within a given sample by the total number of reads. As a final precaution against contaminants and sequencing errors, diet mOTUs with RRA <0.1% [40,44] and nematode mOTUs with RRA <0.2% per sample [7] were removed, as were empty mOTUs/ASVs (those containing no reads across all samples within a dataset).

The final diet dataset comprised 1,322 fecal samples, within which 213 plant taxa from 54 families were detected [40,44]. The final microbiome dataset comprised 509 fecal samples, within which were 29,308 bacterial ASVs from at least 35 phyla [40]. The final nematode dataset comprised 281 samples, within which were 96 strongylid mOTUs from at least 5 families [7].

*Determining nematode characteristics*

Because nematode size and feeding habits might modulate their interactions with hosts and other gut community members [2–4,39], we compiled data on these traits from published sources (**Table S2**). For nematode mOTUs not resolved to species, we used ‘BLAST’ [103] to identify the most plausible species, recording the top ‘BLAST’ hit (by percent identity) or all equally well-matched species. We then determined body lengths and feeding habits for all definitive and plausible species via targeted Google Scholar searches. When lengths were reported as ranges (which was often the case, due to nematode sexual dimorphism), we used the mean; for mOTUs with multiple plausible species matches, we averaged across species. We also categorized nematodes into two feeding habits: *blood-feeders* (nematodes which attach to the gut lining and feed on host blood) and *breachers* (nematodes which encyst in the gut wall, then feed on ingesta upon re-emergence). In total, we estimated lengths for 83 and feeding habits for 95 of 96 mOTUs.

*Assessing diversity and abundance relationships*

For each sample, we calculated taxonomic richness and Shannon diversity (hereafter, ‘diversity’) within each dataset (diet, nemabiome, microbiome, and pathogenic bacteria). To further interrogate compositional effects, we extracted each sample’s position along the first axis of each PCoA ordination. For diet data, we calculated total relative read abundance (RRA) of Poaceae (grasses), reflecting the primary axis of dietary differentiation for savanna herbivores [21,44,45].

For samples with data on putative pathogenic bacteria, we calculated putative pathogenic bacteria species richness (the number of taxa annotated by 16SPIP as having pathogenic potential within a sample), total putative pathogenic bacteria RRA (the summed RRA of all putative pathogenic bacteria taxa detected in a sample), and total putative non-pathogenic bacteria RRA (1 ‒ total putative pathogenic bacteria RRA). Because total putative pathogenic bacteria RRA and putative pathogenic bacteria species richness are likely related due to compositionality constraints, we calculated three additional variables: (1) standardized putative pathogenic bacteria species richness (dividing putative pathogenic bacteria species richness by total putative pathogenic bacteria RRA); (2) average putative pathogenic bacteria RRA (dividing total putative pathogenic bacteria RRA by putative pathogenic bacteria species richness); and (3) average putative non-pathogenic bacteria RRA (dividing total putative non-pathogenic bacteria RRA by putative non-pathogenic bacteria species richness [total microbiome species richness ‒ putative pathogenic bacteria species richness]).

For samples with qPCR data, we estimated nematode abundance as 40 ‒ *C_t_*; abundances < 5 were considered nematode-absent [7]. From nematode compositional data, we calculated total RRA of both blood-feeding and breaching nematodes as well as average nematode length:

average nematode length = $\frac{\sum(RRA*mean length)}{Total RRA of nematodes with length estimates}$

We then computed species-level means for all variables. We included nematode prevalence (calculated as the proportion of nematode qPCR-positive samples per species), species body mass (from COMBINE [58]), and domestication status as species-level predictors.

*Weighting factors predictive of diet, microbiome, and pathogenic microbiome composition*

The same MRM analytical framework employed to assess predictors of nemabiome composition was also applied to the diet, overall microbiome, and putative pathogenic microbiome datasets. For these analyses, we additionally derived an aphylogenetic version of nemabiome dissimilarity using the same residual-based approach described in the main text for aphylogenetic microbiome dissimilarity. In diet models, either phylogenetic or aphylogenetic versions of both microbiome and nemabiome dissimilarities were included together within a given model, to maintain consistency within models. In analyses of overall microbiome and potentially pathogenic microbiome dissimilarity, other bacterial distance matrices were excluded from the predictor set, to avoid circularity.

**SUPPLEMENTARY LITERATURE CITED**

91.	Gill BA, Musili PM, Kurukura S, Hassan AA, Goheen JR, Kress WJ, Kuzmina M, Pringle RM, Kartzinel TR. 2019 Plant DNA-barcode library and community phylogeny for a semi-arid East African savanna. *Molecular Ecology Resources* **19**, 838–846. (doi:10.1111/1755-0998.13001)

92.	Pansu J, Guyton JA, Potter AB, Atkins JL, Daskin JH, Wursten B, Kartzinel TR, Pringle RM. 2019 Trophic ecology of large herbivores in a reassembling African ecosystem. *Journal of Ecology* **107**, 1355–1376. (doi:10.1111/1365-2745.13113)

93.	Boyer F, Mercier C, Bonin A, Le Bras Y, Taberlet P, Coissac E. 2016 obitools: a unix-inspired software package for DNA metabarcoding. *Molecular Ecology Resources* **16**, 176–182. (doi:10.1111/1755-0998.12428)

94.	Callahan BJ, McMurdie PJ, Rosen MJ, Han AW, Johnson AJA, Holmes SP. 2016 DADA2: High-resolution sample inference from Illumina amplicon data. *Nature Methods* **13**, 581–583. (doi:10.1038/nmeth.3869)

95.	DeSantis TZ *et al.* 2006 Greengenes, a chimera-checked 16S rRNA gene database and workbench compatible with ARB. *Appl Environ Microbiol* **72**, 5069–5072. (doi:10.1128/AEM.03006-05)

96.	Bolyen E *et al.* 2019 Reproducible, interactive, scalable and extensible microbiome data science using QIIME 2. *Nat Biotechnol* **37**, 852–857. (doi:10.1038/s41587-019-0209-9)

97.	Kanz C *et al.* 2005 The EMBL Nucleotide Sequence Database. *Nucleic Acids Research* **33**, D29–D33.

98.	Ficetola GF, Coissac E, Zundel S, Riaz T, Shehzad W, Bessière J, Taberlet P, Pompanon F. 2010 An In silico approach for the evaluation of DNA barcodes. *BMC Genomics* **11**, 434. (doi:10.1186/1471-2164-11-434)

99.	Avramenko RW, Redman EM, Lewis R, Bichuette MA, Palmeira BM, Yazwinski TA, Gilleard JS. 2017 The use of nemabiome metabarcoding to explore gastro-intestinal nematode species diversity and anthelmintic treatment effectiveness in beef calves. *International Journal for Parasitology* **47**, 893–902. (doi:10.1016/j.ijpara.2017.06.006)

100. Mercier C, Boyer F, Bonin A, Coissac E. 2013 SUMATRA and SUMACLUST: fast and exact comparison and clustering of sequences. In *Programs and Abstracts of the SeqBio 2013 Workshop*, pp. 27–29.

101. Van Dongen S. 2008 Graph Clustering Via a Discrete Uncoupling Process. *SIAM J. Matrix Anal. Appl.* **30**, 121–141. (doi:10.1137/040608635)

102. McKnight DT, Huerlimann R, Bower DS, Schwarzkopf L, Alford RA, Zenger KR. 2019 microDecon: A highly accurate read-subtraction tool for the post-sequencing removal of contamination in metabarcoding studies. *Environmental DNA* **1**, 14–25. (doi:10.1002/edn3.11)

103. Altschul SF, Gish W, Miller W, Myers EW, Lipman DJ. 1990 Basic local alignment search tool. *J Mol Biol* **215**, 403–410. (doi:10.1016/S0022-2836(05)80360-2)

**SUPPLEMENTARY FIGURES**

**Figure S1.** **Within-species compositional variation.** PCoA ordination plots show Bray-Curtis dissimilarity in (a) nematode, (b) diet, (c) overall bacterial, and (d) putative pathogenic bacterial community composition as reconstructed from DNA metabarcoding of herbivore fecal samples. Each point represents individual fecal samples. Ellipses represent bivariate elliptical 95% probability regions (*i.e.*, regions expected to contain 95% of points) for each species. Point and ellipse colors correspond to the herbivore species. Statistics from perMANOVAs evaluating the significance of herbivore species identity in structuring community composition are shown in each panel. In all cases, samples clustered strongly by herbivore species, confirming results from prior work [7,40,44].

**Figure S2. Pairwise cooccurrence network of herbivore species, diet items, nematodes, and putative pathogenic bacteria.** The full network is depicted in (a); more than half of links (449/855; 52.5%) were between dietary plants and were therefore removed to facilitate network visualization. In (b) are links between herbivore species and other network members. In (c) are links between diet plants and gut community members. In (d) are links between gut community members only. Networks in (b–d) are split into positive (only positive associations, on the left) and negative (only negative associations, on the right) modules; positive links between taxa (where taxa co-occur significantly more than would be expected by chance) are colored *blue* while negative links between taxa (where taxa co-occur significantly less than would be expected by chance) are colored *red*. Nodes are uniquely numbered, and names associated with each number are listed to the right of the network. Node shapes and colors correspond to the node type: *teal diamonds* (1–11) correspond to host species, *green circles* (12–95) correspond to diet plant mOTUs, *yellow squares* (96–127) correspond to nematode species, and *blue circles* (128–156) correspond to putative pathogenic bacteria species.

**Figure S3. Abundance of putative pathogenic bacteria increases with diet richness but declines with microbiome diversity.** (a–b) Putative pathogenic bacterial richness increased with increasing diet richness, as did (c–d) putative pathogenic bacterial relative abundance. However, (e–f) putative pathogenic bacterial relative abundance declined with increasing microbiome diversity. Panels (a, c, e) depict species-level relationships, such that points correspond to within-species means, while panels (b, d, f) depict the corresponding sample-level relationships, such that each point represents a single sample. *Points* are colored by species identity. *Blue regression lines* correspond to positive relationships, while *red regression lines* correspond to negative relationships. *Solid lines* in (a, c, e) correspond to aphylogenetic regressions (LM), while *dashed lines* correspond to equivalent phylogenetic regressions (phyloLM). *Thinner colored lines* in (b, d, f) correspond to linear mixed-effects models (LMM) including a random intercept for species and are thus colored by species identity. Statistics from all models are shown in the corresponding panels.
